# Genetic characterisation of African swine fever virus from 2017 outbreaks in Zambia: Identification of *p72* genotype II variants in domestic pigs

**DOI:** 10.4102/ojvr.v85i1.1562

**Published:** 2018-06-26

**Authors:** Edgar Simulundu, Yona Sinkala, Herman M. Chambaro, Andrew Chinyemba, Frank Banda, Lynnfield E. Mooya, Joseph Ndebe, Simbarashe Chitanga, Chitwambi Makungu, Gift Munthali, Paul Fandamu, Ayato Takada, Aaron S Mweene

**Affiliations:** 1 Department of Disease Control, University of Zambia, Zambia; 2 Department of Veterinary Services, Ministry of Fisheries and Livestock, Zambia; 3 Department of Biomedical Sciences, University of Zambia, Zambia; 4 Division of Global Epidemiology, Hokkaido University Research Center for Zoonosis Control, Japan

## Abstract

African swine fever (ASF) is a contagious haemorrhagic disease associated with causing heavy economic losses to the swine industry in many African countries. In 2017, Zambia experienced ASF outbreaks in Mbala District (Northern province) and for the first time in Isoka and Chinsali districts (Muchinga province). Meanwhile, another outbreak was observed in Chipata District (Eastern province). Genetic analysis of part of the *B646L* gene, *E183L* gene, *CP204L* gene and the central variable region of the *B602L* gene of ASF virus (ASFV) associated with the outbreaks in Mbala and Chipata districts was conducted. The results revealed that the ASFV detected in Mbala District was highly similar to that of the Georgia 2007/1 isolate across all the genome regions analysed. In contrast, while showing close relationship with the Georgia 2007/1 virus in the *B646L* gene, the ASFV detected in Chipata District showed remarkable genetic variation in the rest of the genes analysed. These results suggest that the Georgia 2007/1-like virus could be more diverse than what was previously thought, underscoring the need of continued surveillance and monitoring of ASFVs within the south-eastern African region to better understand their epidemiology and the relationships between outbreaks and their possible origin.

## Introduction

African swine fever virus (ASFV) causes African swine fever (ASF), an acute, contagious and devastating haemorrhagic disease that affects swine. Because of its high mortality rates (up to ≈100%), serious socio-economic impact, high capacity for transboundary dissemination and lack of an effective vaccine or treatment, ASF is considered to be one of the most challenging animal diseases to contend with (Costard et al. 2009).

Historically, ASF was first described in Kenya in 1921 and is considered to be endemic in many African countries (Penrith et al. 2013). Indeed, reports of outbreaks in at least 26 African countries during 2009–2011 testify to the disease’s continued eminent presence and the threat it poses to the pig industry in sub-Saharan Africa (Penrith et al. 2013). Based on sequence analysis of the variable C-terminus of the *B646L* gene encoding the *p72* capsid protein, ASFV field strains are genetically categorised into 24 genotypes (Achenbach et al. 2016; Bastos et al. 2003; Quembo et al. 2018). Traditionally, while all the genotypes have been circulating within south-eastern Africa, only genotype I has been reported from and predominates in West Africa (Brown et al. 2017). Moreover, genotype I ASFV was introduced into Europe, South America and the Caribbean, but has only remained endemic in Sardinia (Italy) (Costard et al. 2009). However, the global ASF situation changed dramatically following the introduction of a highly virulent genotype II ASFV, which was circulating in south-eastern Africa, into Georgia in 2007 (Costard et al. 2009; Rowlands et al. 2008). The virus spread rapidly and eventually advanced into eastern Europe, a situation that has raised serious concerns over the global pig industry (Vergne et al. 2017).

In recent years, Zambia has suffered frequent sporadic ASF outbreaks in almost all its provinces, which have been associated with multiple genotypes (Simulundu et al. 2017, 2018; Thoromo et al. 2015). In 2017, ASF outbreaks occurred in Mbala (Northern province), Isoka and Chinsali (Muchinga Province) districts. Furthermore, an outbreak was also observed in May 2017 in Chipata District (Eastern province). With the aim of increasing knowledge about the epidemiology of ASF, findings on genetic analysis of distinct genome regions of ASFVs associated with outbreaks in Mbala and Chipata districts are reported.

## Materials and methods

On 26 April 2017, an ASF outbreak in Zambia, which occurred in Mbala District in free-range domestic pigs, was reported (World Organisation for Animal Health [OIE] 2017a). The affected village had 48 pigs in which 15 cases and 11 deaths (73.3% case fatality rate) were recorded. The disease was observed in Chinsali and Isoka districts for the first time (OIE 2017b). In these two districts, a total of 1895 cases with 1891 deaths (99.8% case fatality rate) were reported. The ASF outbreak in Chipata District involved a farmer whose 28 indigenous pigs were affected and 20 had died (71.4% case fatality rate).

For laboratory diagnosis, DNA was extracted from tissue homogenates on samples (kidneys, lymph nodes, tonsils and spleens) collected from pigs that died from the disease. Molecular diagnosis was conducted by polymerase chain reaction (PCR) using the ASF diagnostic primer set PPA1/PPA2 (Agüero et al. 2003; Yabe et al. 2015). For genetic characterisation, international standardised procedures for studying the molecular epidemiology of ASF (OIE 2013) were applied on PCR-positive samples. Specifically, the C-terminal end of the *p72* (*B646L*) gene, *p54* (*E183L*) gene, *p30* (*CP204L*) gene and the central variable region (CVR) within the *B602L* gene were amplified using the primer pairs p72U/p72D, PPA722/PPA89, p30-F/p30-R and CVR1/CVR2 or ORF9L-F/ORF9L-R, respectively (Simulundu et al. 2017, 2018). Purified PCR products were sequenced directly via the Sanger technology using a 3500 Genetic Analyzer (Applied Biosystems, Foster City, CA, USA). The sequences were deposited in GenBank (accession no. LC322009-LC322016). Two ASFVs, designated ZAM/2017/Mbala/1 and ZAM/2017/Chipata/1, were genetically characterised. Evolutionary relationships were inferred using the neighbour-joining method, while evolutionary distances were computed using the *p*-distance method. The genetic trees were generated using MEGA6 software, version 6.06 (Tamura et al. 2013).

## Results and discussion

Nucleotide sequence comparisons using the Basic Local Alignment Search Tool (BLAST) (http://blast.ncbi.nlm.nih.gov/Blast.cgi) revealed that the *p72,* *p54* and *p30* sequences of ZAM/2017/Mbala/1 were 100% identical to those of Georgia 2007/1 (GenBank accession no. AM999764-AM999766). In contrast, BLAST showed that the *p72* and *p30* nucleotide sequences of ZAM/2017/Chipata/1 were 99% – 100% similar to those of ZAM/14/Chipata, which was detected in domestic pigs in the same district (Simulundu et al. 2018). The *p54* nucleotide sequence of ZAM/2017/Chipata/1 showed 100% similarity to that of KLI/88/2, which was found in Petauke District, Eastern province, in 1988 (Simulundu et al. 2017).

**FIGURE 1:**
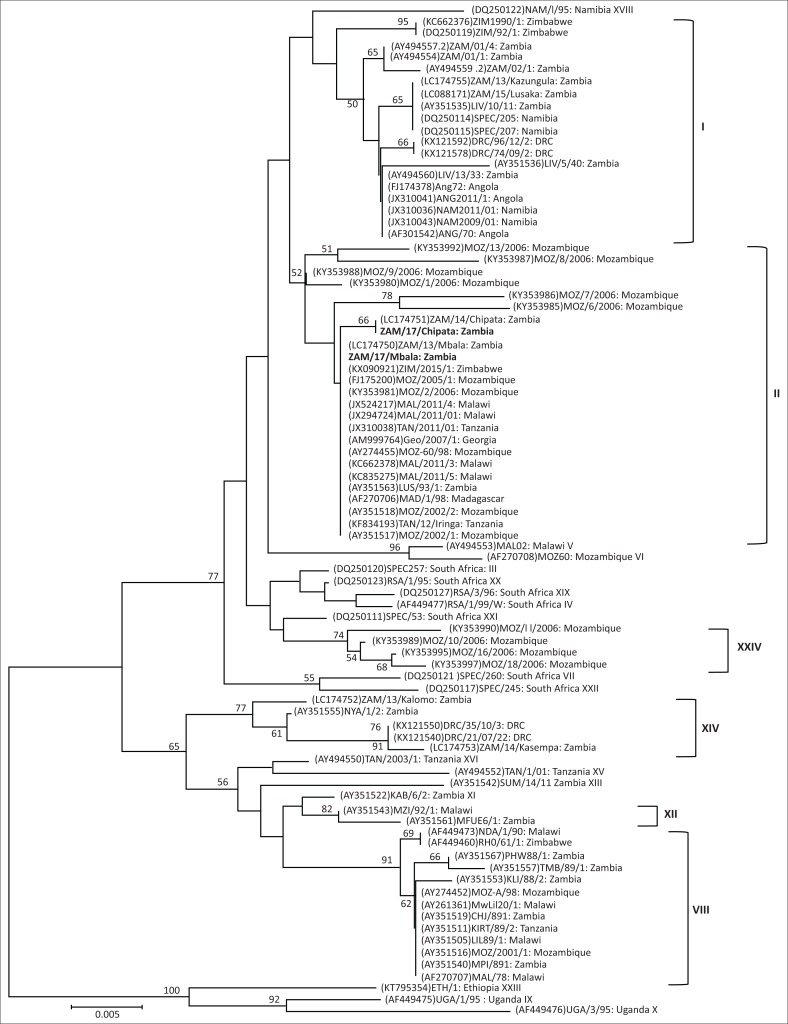
Phylogenetic relationships of the *p72* gene of African swine fever viruses detected in domestic pigs during 2017 in Zambia. The evolutionary relationships were conducted by MEGA6 software using the neighbour-joining method, with evolutionary distances being computed by the *p*-distance method. Numbers at branch nodes indicate bootstrap values ≥ 50%. The GenBank accession numbers of strains included in the analyses are indicated in parenthesis and the *p72* genotypes are shown after the country of origin of the strains or the right bracket. Virus strains characterised in this study are in bold. Bar, number of substitutions per site.

**FIGURE 2:**
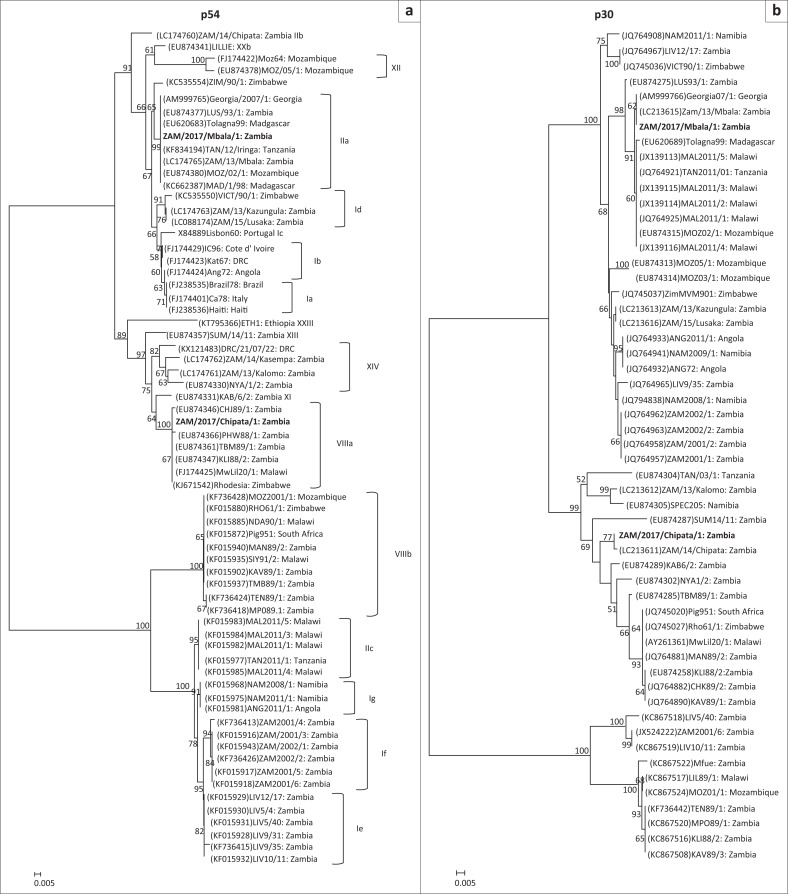
Phylogenetic relationships of the *p54* (a) and *p30* (b) genes of African swine fever viruses detected in domestic pigs during 2017 in Zambia. The evolutionary relationships were conducted by MEGA6 software using the neighbour-joining method, with evolutionary distances being computed by the *p*-distance method. Numbers at branch nodes indicate bootstrap values ≥ 50%. The GenBank accession numbers of strains included in the analyses are indicated in parenthesis and the *p54* genotypes are shown after the country of origin of the strains or the right bracket. Virus strains characterised in this study are in bold. Bar, number of substitutions per site.

Phylogenetic analysis showed that both ZAM/2017/Mbala/1 and ZAM/2017/Chipata belonged to *p72* genotype II (Figure 1). In contrast, the *p54* phylogeny revealed that ZAM/2017/Mbala/1 belonged to genotype IIa while ZAM/2017/Chipata/1 grouped under genotype VIIIa (Figure 2a). Thus, the *p54* genetic tree showed that *p72* genotype II viruses were separated into distantly related genotypes IIa, IIb (solely comprising ZAM/14/Chipata) and IIc (composed of ASFVs associated with outbreaks in Tanzania and Malawi in 2011) and the Zambian virus that fell into genotype VIIIa (Figure 2a). Consistent with the *p72* and *p54* evolutionary relationships, ZAM/2017/Mbala/1 was closely related to ZAM/13/Mbala and Georgia 2007/1 viruses in the *p30* tree (Figure 2b). In contrast, ZAM/2017/Chipata/1 and ZAM/14/Chipata formed a distinct group that was distantly related to the Georgia 2007/1-like viruses (Figure 2b).

While amino acid sequence analysis of the CVR revealed that the sequence of ZAM/2017/Mbala/1 possessed ten copies (BNDBNDBNAA) of tetramer repeats that were 100% similar to those circulating in eastern Europe since 2007, the sequence of ZAM/2017/Chipata/1 was 100% identical to that of ZAM/14/Chipata, with 14 copies (BNABNDBTDBNAAG) (Simulundu et al. 2017, 2018).

Notably, while the case fatality rate in Mbala District was lower (73.3%) than that in Isoka and Chinsali (99.8%), it was comparable to that observed in Chipata (71.4%) where ASF is endemic. Perhaps this could be explained by the fact that since 2013 Mbala District has had prior experience with ASF associated with genotype II virus (Simulundu et al. 2017, 2018) and thus some pigs may have developed some level of immunity. Moreover, antibodies to ASFV were detected in the serum of pigs in the district by ELISA (OIE 2017a), a finding which suggests possible persistent circulation of the virus in the area. Meanwhile, Isoka and Chinsali districts were affected by ASF for the first time and possibly the naïve pig population was highly susceptible to ASFV infection.

In Zambia, *p72* genotype II ASFV similar to Georgia 2007/1 isolate was first detected in 1993 in Lusaka Province (Simulundu et al. 2017). About two decades later, this viral genotype was associated with outbreaks in Mbala District, Northern province, probably as a result of introduction from neighbouring Tanzania and not from Eastern province (Simulundu et al. 2018). Furthermore, in 2014, a genotype II variant was associated with an outbreak in Chipata District (Simulundu et al. 2017, 2018). Along with the identification of yet another genotype II variant, ZAM/2017/Chipata/1, and the observation that ASFVs associated with outbreaks in 2011 in Tanzania and Malawi belonged to *p54* genotype IIc, which is different from that of Georgia 2007/1 (genotype IIa) (Figure 2a), these findings suggest that in the south-eastern African region, these viruses could be diverse, probably because of the presence of a sylvatic cycle and long-term endemicity. The *p30* phylogeny provided further evidence on the genetic diversity of *p72* genotype II viruses as ZAM/2017/Chipata/1 and ZAM/14/Chipata belonged to a group that was phylogenetically distinct from that of Georgia 2007/1-like viruses (Figure 2b).

Meanwhile, reminiscent of the eastern Europe situation, the geographical extent of genotype II ASFV in south-eastern Africa appears to be expanding as this virus has been detected in Madagascar, Malawi, Mauritius, Mozambique, Tanzania, Zambia and Zimbabwe (Simulundu et al. 2017; van Heerden et al. 2017). Historically, genotype II ASFV has been circulating in Mozambique and Zambia for about 20–25 years (Bastos et al. 2004; Boshoff et al. 2007; Penrith et al. 2007; Simulundu et al. 2017). This genotype was introduced in Madagascar in 1997, most likely from Mozambique. Tanzania had its first experience with ASF outbreaks associated with genotype II virus during 2010–2012, possibly as a result of spread from Malawi (Misinzo et al. 2012). The virus appears to have become endemic in domestic pigs in Tanzania. This virus spread to Mauritius in 2007, probably from Madagascar, and was eradicated in 2008 (Lubisi et al. 2009). The identification of genotype II ASFV in domestic pigs in Zimbabwe in 2015 was traced to outbreaks in an endemic region in Mozambique (van Heerden et al. 2017). More recently, *p72* genotype II ASFVs were detected in soft tick reservoir hosts in Mozambique, providing evidence for the first time of a possible sylvatic origin of these viruses in south-eastern Africa (Quembo et al. 2018). These observations underscore the need of continued surveillance and monitoring of ASFVs within the south-eastern African region to better understand their epidemiology and the relationships between outbreaks and their possible origin.

## Conclusion

The present study investigated the molecular epidemiology of ASF outbreaks that occurred in 2017 in Northern, Muchinga and Eastern provinces of Zambia. Genetic analysis showed that the outbreaks in the Northern region were caused by genotype II ASFV, which was highly similar to the Georgia 2007/1 isolate. Although belonging to genotype II, the ASFV associated with the outbreak in Eastern province was genetically diverse, suggesting that these outbreaks were not related. Overall, this study supports the idea that genotype II ASFV circulating in south-eastern Africa is genetically diverse, probably because of the long-term endemicity in domestic pigs and a possible sylvatic origin.
